# Supplementary material for: Unravelling the drivers of marine biodiversity across the Phanerozoic
Source: Nat Commun. 2025 Sep 26;16:8498. doi: 10.1038/s41467-025-63428-9 (PMC12475154; doi:10.1038/s41467-025-63428-9)
Supplement: Supplementary file 1 — Supplementary Information [file 41467_2025_63428_MOESM1_ESM.pdf]

Supplementary Information

Unravelling the drivers of marine biodiversity across the Phanerozoic

Alexis Balembois[1*], Alexandre Pohl[2], Bertrand Lefebvre[3], Thomas Servais[4], Dan J. Lunt[5], Paul J. Valdes[5], Grégory Beaugrand[1*]

[1] Univ. Littoral Côte d'Opale, CNRS, Univ. Lille, UMR 8187 LOG, F-62930 Wimereux, France

[2] Biogéosciences, UMR 6282 CNRS, Université de Bourgogne, 6 boulevard Gabriel, 21000 Dijon, France

[3] Univ Lyon, Univ Lyon 1, ENSL, CNRS, LGL-TPE, F-69622, Villeurbane, France

[4] Univ. Lille, CNRS, ULR 8198-Evo-Eco-Paleo, F-59000 Lille, France

[5] School of Geographical Sciences, University of Bristol, Bristol BS8 1SS, UK

* Corresponding authors: alexis.balembois@univ-littoral.fr, gregory.beaugrand@cnrs.fr

Variables	**Modelled species richness (no speciation)**	**Modelled species richness (with allopatric speciation)**	**Marine area around continents**	**Continental fragmentation index**	**Fossil species richness index**	**Latitudinal Continental Index (LCI)**	**LBG-weighted LCI**
**Fossil species richness index**	0.08	**0.73**	**0.75**	0.57	**1**	**0.76**	***0.66***
**Biodiversity after Alroy *et al*.**[1]	0.21	**0.74**	**0.72**	***0.58***	**0.89**	**0.70**	**0.68**
**Biodiversity after a recently-updated version of the Sepkoski's database**	0.07	**0.70**	**0.72**	0.56	**0.95**	**0.73**	***0.60***
**Biodiversity after PBDB**	0.00	*0.38*	**0.44**	0.34	**0.58**	**0.45**	0.34
**Biodiversity after Sepkoski *et al*.**[2]	0.06	**0.70**	**0.73**	0.55	**0.97**	**0.74**	***0.62***
**Biodiversity after Zaffos *et al*.**[3]	-0.05	***0.55***	**0.60**	0.40	**0.90**	**0.63**	***0.53***
**Continental-shelf area**	0.40	**0.90**	**1**	***0.74***	**0.75**	**0.97**	**0.93**
**Continental fragmentation index**	0.21	**0.80**	***0.74***	**1**	0.57	0.63	***0.71***
**Latitudinal Continental Index (LCI)**	0.35	**0.83**	**0.97**	0.63	**0.76**	**1**	**0.91**
**LBG-weighted LCI**	0.54	**0.90**	**0.93**	***0.71***	***0.6***	**0.91**	**1**

Supplementary Table 1. Linear correlations. Significant correlation ($p_{ACF} \leq 0.05$ after accounting for temporal autocorrelation, degree of freedom = 107) have been indicated in bold font and almost significant correlation ($0.05 < P_{ACF} \leq 0.1$ after autocorrelation correction, degree of freedom = 107) have been indicated in bold font and italic. Continental fragmentation index is after ref.[3].

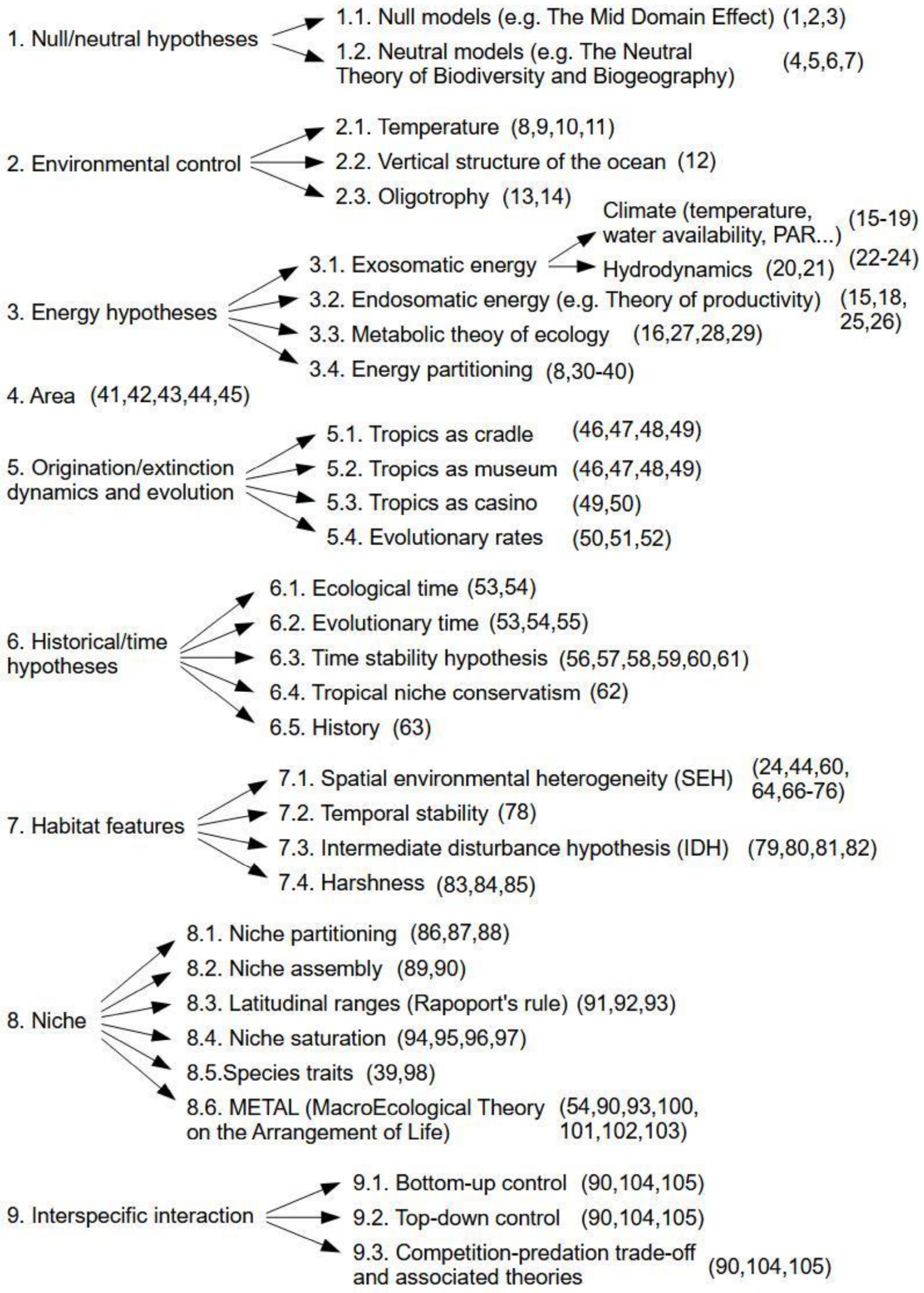

Supplementary Figure 1. Hypotheses or theories proposed to explain global biodiversity patterns, including the latitudinal biodiversity gradient (LBG). Numbers in parentheses are references according to Supplementary Note 1. See Supplementary Note 1 for explanations.

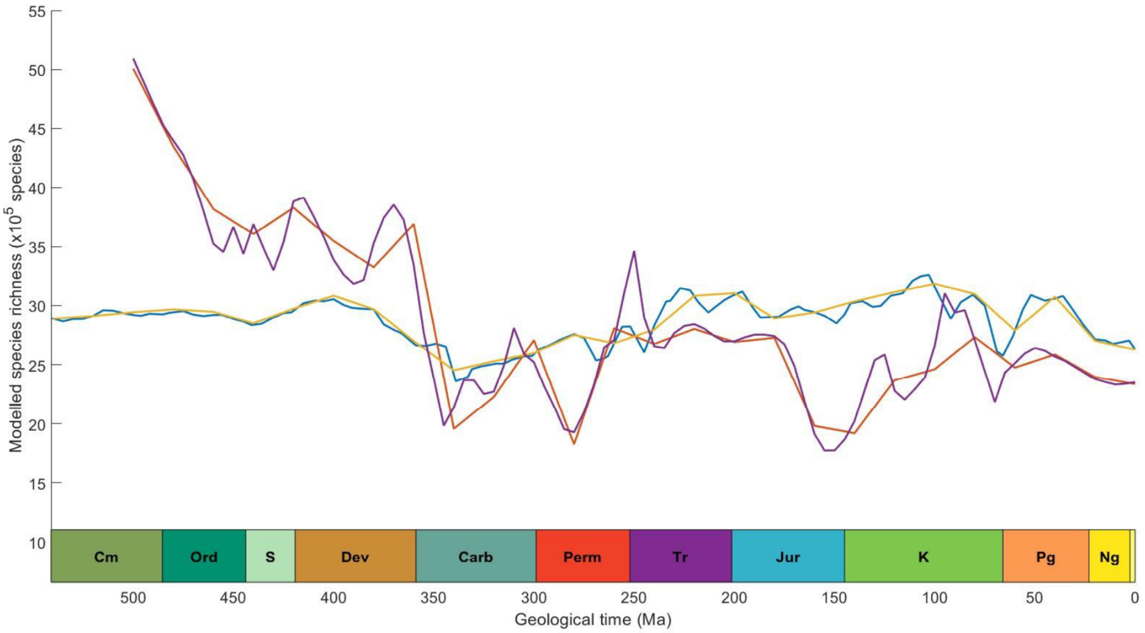

Supplementary Figure 2. Tropical sea-surface temperatures. Mean tropical (30 °S to 30 °N) SSTs in the HadCM3 simulations of ref.[4] (blue line, this study) and mean tropical (30 °S to 30 °N) SSTs in the cGENIE simulations designed to reproduce this long-term SST trend (yellow line). Tropical SSTs reconstructed by ref.[5] based on oxygen isotopes (purple line) and mean tropical (30 °S to 30 °N) SSTs in the cGENIE simulations designed to reproduce this long-term SST trend[5] (orange line). The differences between the blue and yellow lines, and the purple and orange lines, mostly arise from differences in the time resolution of each couples of curves – respectively 109, 28, 101 and 26 time slots being used. Cm: Cambrian; Ord: Ordovician; S: Silurian; Dev: Devonian; Carb: Carboniferous; Perm: Permian; Tr: Triassic; Jur: Jurassic; K: Cretaceous; Pg: Paleogene; Ng: Neogene. Ma: million years ago.

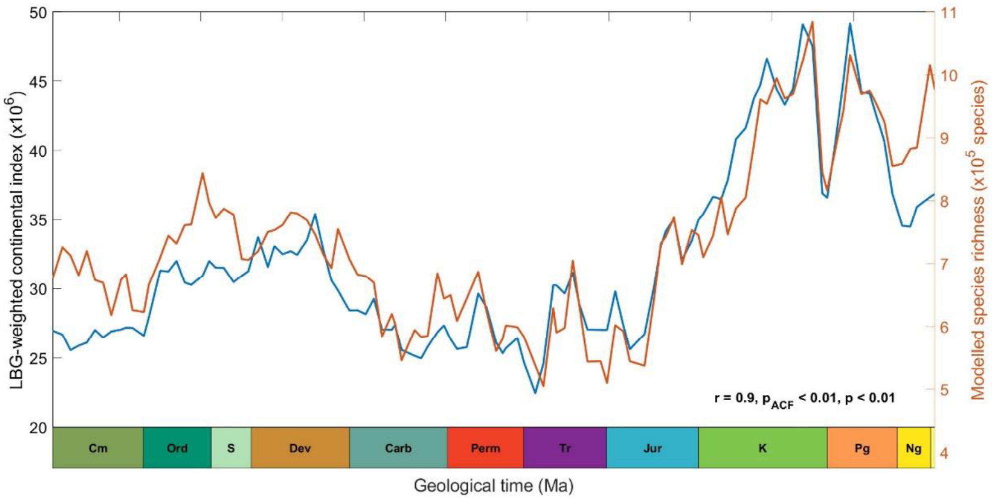

Supplementary Figure 3. Comparison between long-term changes in LGB-weighted LCI and HadCM3-based modelled species richness. The correlation coefficient r, and its probability before (p) and after (p_{ACF}, ACF for autocorrelation function) accounting for temporal autocorrelation are provided in the bottom-right corner. Cm: Cambrian; Ord: Ordovician; S: Silurian; Dev: Devonian; Carb: Carboniferous; Perm: Permian; Tr: Triassic; Jur: Jurassic; K: Cretaceous; Pg: Paleogene; Ng: Neogene. Ma: million years ago.

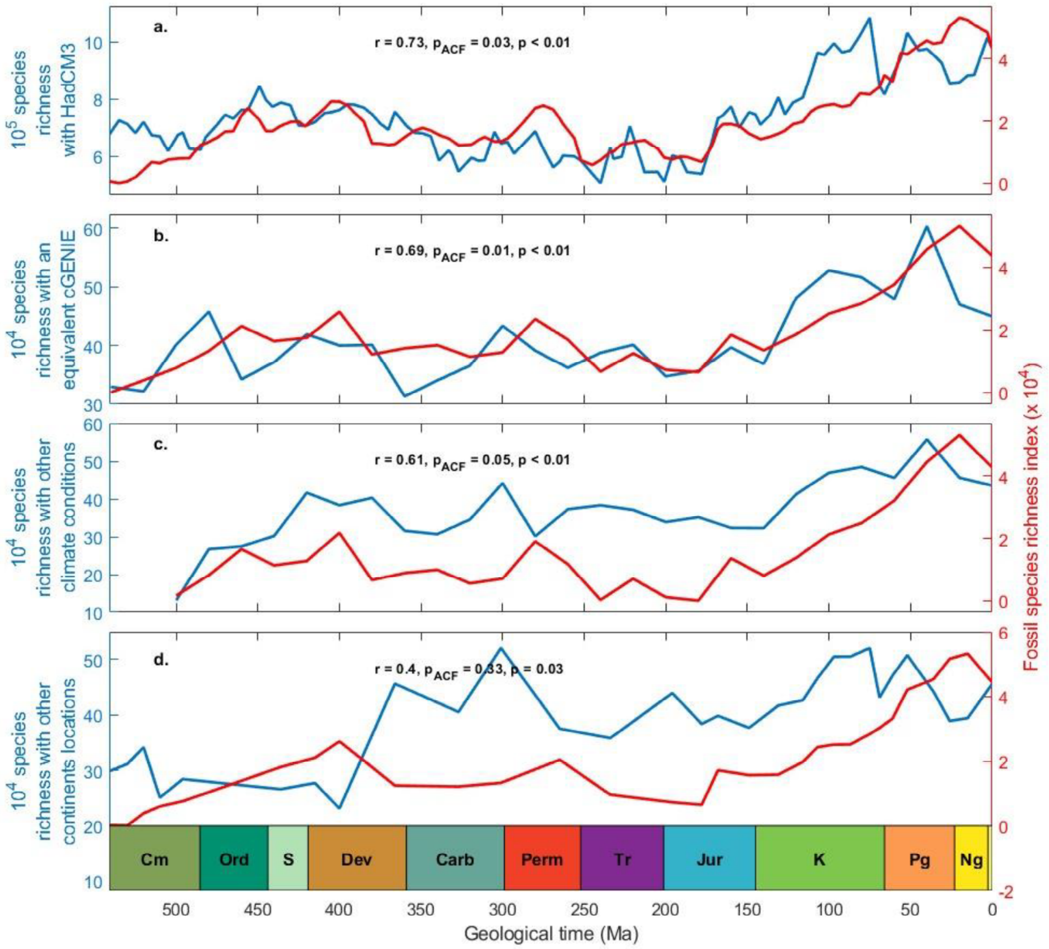

Supplementary Figure 4. Sensitivity of long-term marine biodiversity trends to climate model, SST scenario and palaeogeographical reconstruction. a Long-term changes in the fossil species richness index *versus* global biodiversity simulated using the HadCM3 SSTs of ref.[4]. The HadCM3 simulations use the Phanerozoic continental reconstructions of ref.[6].This panel is identical to Fig. 3b. **b** As per panel a but based on cGENIE simulations designed to reproduce the long-term mean tropical (30 °S to 30 °N) SST trend of the HadCM3 simulations (see Supplementary Fig. 2), also using the continental reconstructions of ref.[6]. **c** As per panel b but based on cGENIE simulations designed to reproduce the long-term SST trend of ref.[5] (see Supplementary Fig. 2). In this alternative SST scenario, SSTs are higher during the early Palaeozoic (Cambrian and Ordovician). The continental reconstructions of ref.[6] are used. **d** As per panel b (cGENIE simulations designed to reproduce the long-term SST trend of the HadCM3 simulations) but using an alternative set of palaeogeographical reconstructions[7] (Methods). The coefficient r for the correlation with the fossil species richness index, and its probability before (p) and after (p_{ACF}, ACF for autocorrelation function) accounting for temporal autocorrelation are provided in every panel. Cm: Cambrian; Ord: Ordovician; S: Silurian; Dev: Devonian; Carb: Carboniferous; Perm: Permian; Tr: Triassic; Jur: Jurassic; K: Cretaceous; Pg: Paleogene; Ng: Neogene. Ma: million years ago.

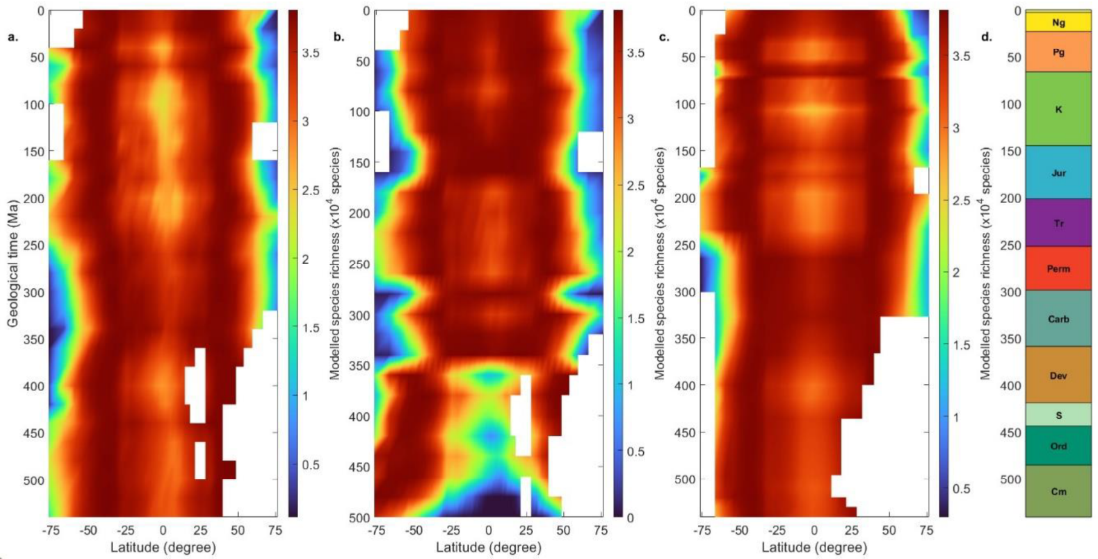

Supplementary Figure 5. Sensitivity of long-term changes in LBGs to climate model, SST scenario and palaeogeographical reconstruction. a. Long-term changes in latitudinal species richness based on cGENIE simulations designed to reproduce the long-term SST trend of the HadCM3 simulations of ref.[51] (see Supplementary Fig. 2), also using the continental reconstructions of ref.[6]. **b.** As per panel a but based on cGENIE simulations designed to reproduce the long-term SST trend of ref.[5] (see Supplementary Fig. 2). In this alternative SST scenario, SSTs are higher during the early Palaeozoic (Cambrian and Ordovician). The continental reconstructions of ref.[6] are used. **c** As per panel a (cGENIE simulations designed to reproduce the long-term SST trend of the HadCM3 simulations) but using an alternative set of palaeogeographical reconstructions[7] (Methods). **d.** Geological chart. Cm: Cambrian; Ord: Ordovician; S: Silurian; Dev: Devonian; Carb: Carboniferous; Perm: Permian; Tr: Triassic; Jur: Jurassic; K: Cretaceous; Pg: Paleogene; Ng: Neogene. Ma: million years ago. White latitudinal cells correspond to latitudes and times with no continent.

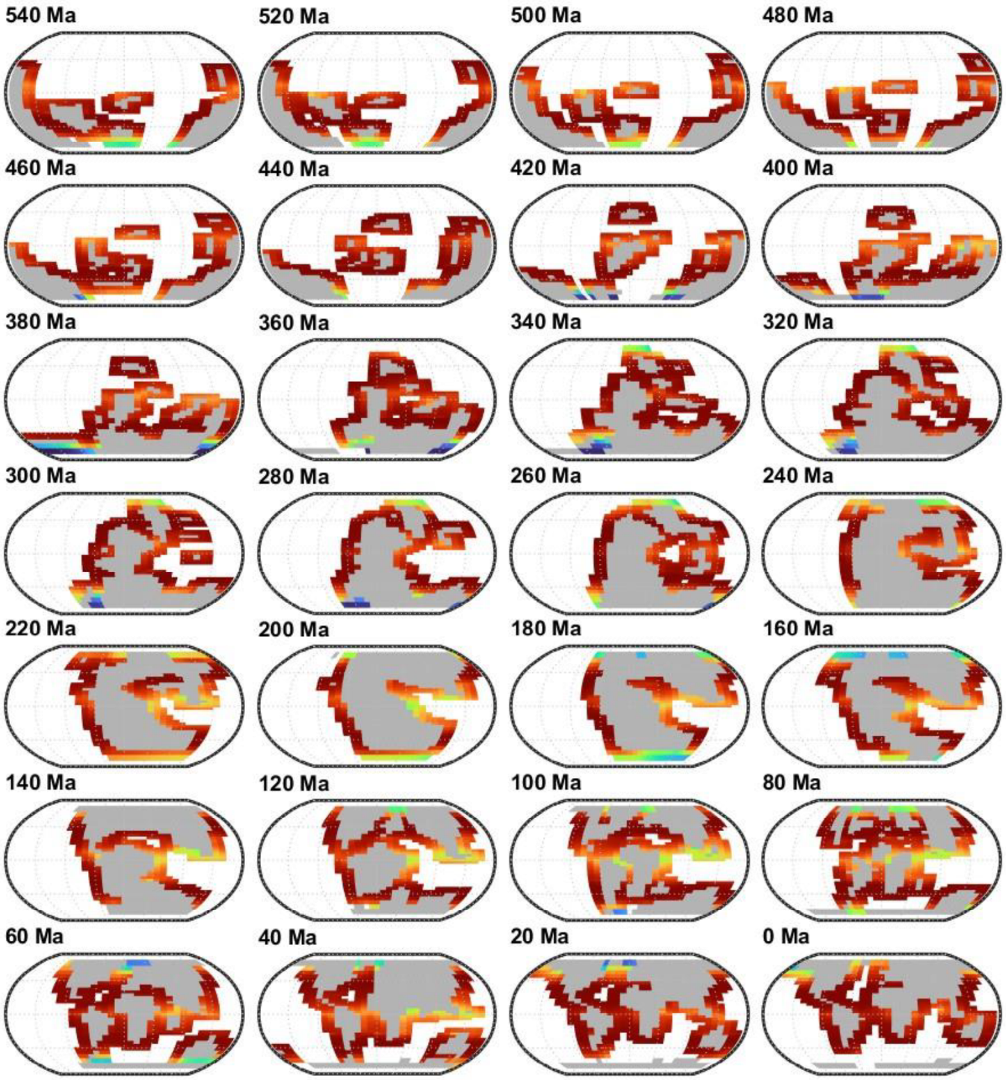

Supplementary Figure 6. Global maps of simulated marine biodiversity during the Phanerozoic based on SSTs simulated using cGENIE. Robinson projection with parallels shown every 30° latitude. Emerged landmasses are shaded gray. As per Fig. 2 but using SSTs simulated using cGENIE instead of HadCM3 (see main text and Methods).

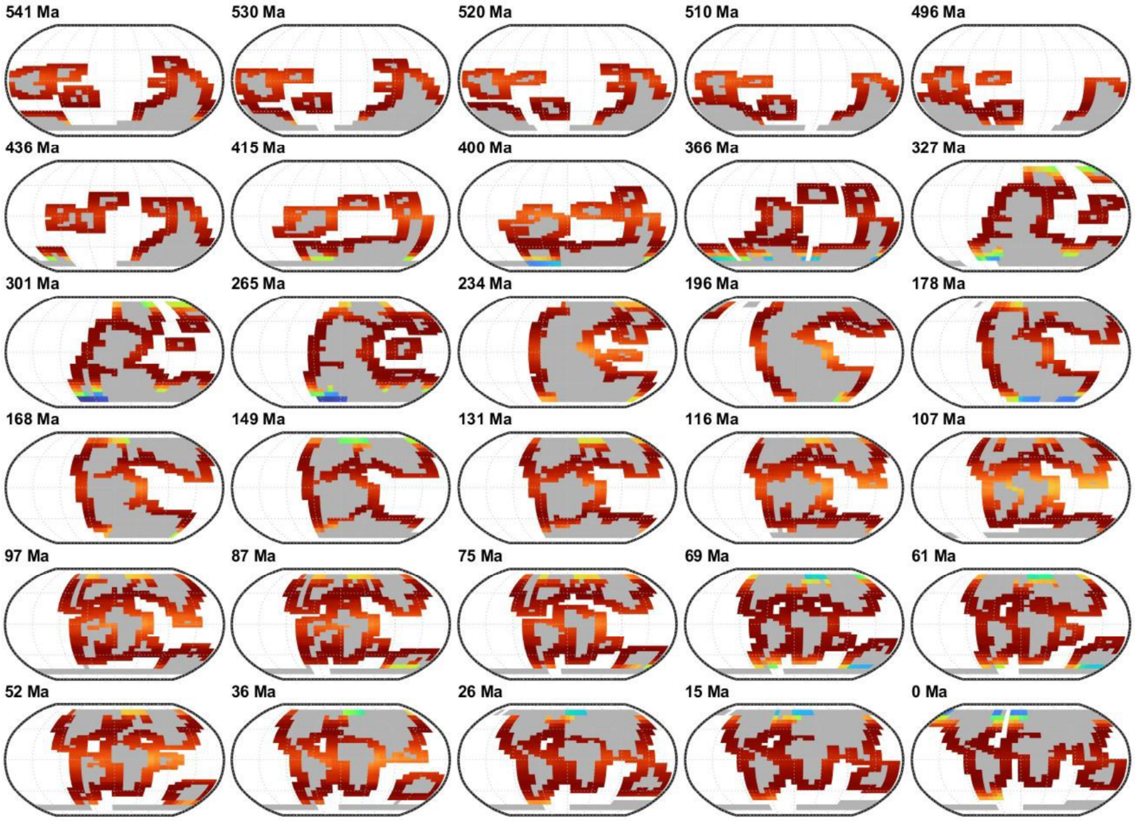

Supplementary Figure 7. Global maps of simulated marine biodiversity during the Phanerozoic based on SSTs simulated using cGENIE and the palaeogeographical reconstructions of ref.[7]**.** Robinson projection with parallels shown every 30° latitude. Emerged landmasses are shaded gray. As per Fig. 2 but using SSTs simulated using cGENIE instead of HadCM3 and an alternative set of Phanerozoic palaeogeographical reconstructions[7] (see main text and Methods).

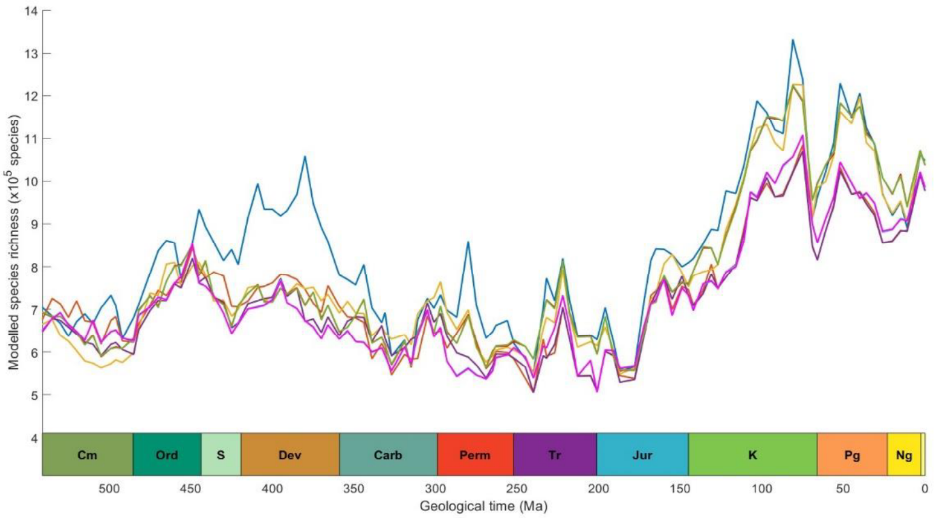

Supplementary Figure 8. Sensitivity to model spatial domain. Simulations run considering marine areas defined as (i) 1 coastal grid cell around continents (blue line), (ii) 2 coastal grid cells around continents (as per Fig. 3b) (orange), (iii) 1 coastal grid cell and a bathymetry threshold of 600 m below sea level (b.s.l.) (yellow), (iv) 2 coastal grid cells and a bathymetry threshold of 600 m b.s.l. (purple), (v) 1 coastal grid cell and a bathymetry threshold of 1000 m b.s.l. (red), (vi) 2 coastal grid cells and a bathymetry threshold of 1000 m b.s.l. (sky blue), (vii) 1 coastal grid cell and a bathymetry threshold of 1500 m b.s.l. (green), (viii) 2 coastal grid cells and a bathymetry threshold of 1500 m b.s.l. (magenta). Several lines are very similar and overlap. Cm: Cambrian; Ord: Ordovician; S: Silurian; Dev: Devonian; Carb: Carboniferous; Perm: Permian; Tr: Triassic; Jur: Jurassic; K: Cretaceous; Pg: Paleogene; Ng: Neogene. Ma: million years ago.

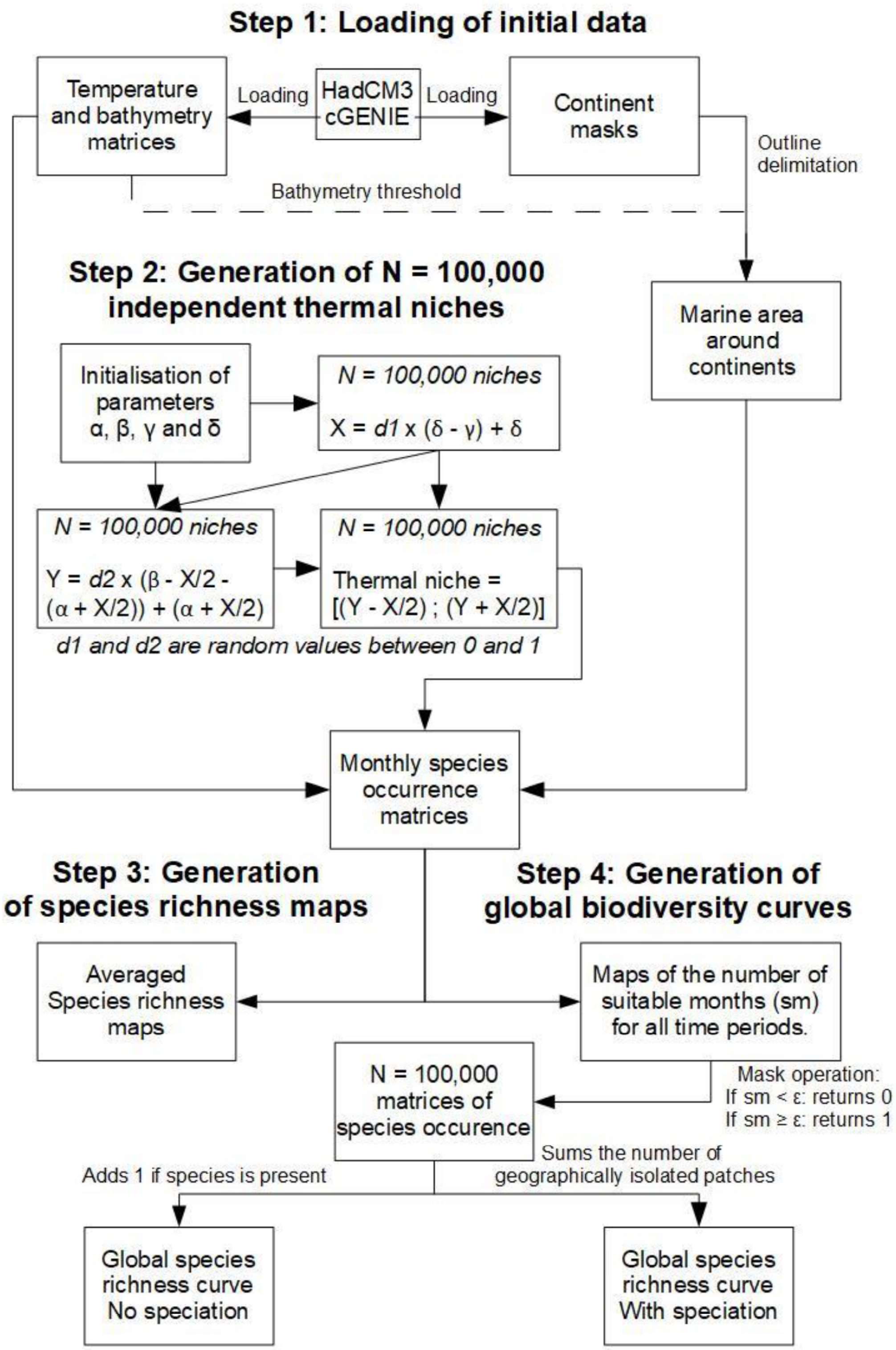

Supplementary Figure 9. Sketch diagram summarizing the four main steps involved in our macroecological model. In Step 1, environmental matrices (i.e. monthly SSTs, continents position, bathymetry) are loaded from palaeoclimatic simulations and marine area around continents is computed (Methods). In Step 2, N = 100,000 independent thermal niches are

generated. First, parameters corresponding to lower (α) and upper (β) temperature limits of organisms, and lower (γ) and upper (δ) degrees of eurythermy (i.e. minimum and maximum range of thermal tolerance), are initialised. Then, two parameters X and Y, being respectively the extent of a given species tolerance range and the centre of its ecological niche are calculated for each niche as: $X = d1 \times (\delta - \gamma) + \delta$ and $Y = d2 \times (\beta - X/2 - (\alpha + X/2)) + (\alpha + X/2)$ with d1 and d2 two random scalar values between 0 and 1 that generate stochasticity. Finally, thermal niches are defined as the range of temperatures between $(Y - X/2)$ and $(Y + X/2)$. In Step 3, ecological niches are projected into a geographical space based on monthly SST data. 12 monthly species richness maps are generated for every time period as a sum of the N = 100,000 species individual absence/presence (0,1) maps and averaged geographical cell per geographical cell to get one global species richness map per time period. Finally, in Step 4, global biodiversity curves are generated, with one global species richness value per time period. First, species presence/absence maps are summed to get N = 100,000 maps of the number of suitable months for each time period. Then, a variable ε, corresponding to a threshold in the minimum number of suitable months for a species to maintain in a given geographical cell, is initialised. N = 100,000 maps of annual species occurrences are generated by transforming previous maps into combinations of 0 when the number of annual suitable months is lower to ε and 1 otherwise. Finally, global species richness curves (i.e. with and without speciation) are generated by summing respectively the number of species occurring in at least one geographical cell or the number of geographically isolated patches for each time period.

SUPPLEMENTARY INFORMATION REFERENCES

1. Alroy, J. *et al.* Phanerozoic trends in the global diversity of marine invertebrates. *Science* **321**, 97–100 (2008).
2. Sepkoski, J. J., Bambach, R. K., Raup, D. M. & Valentine, J. W. Phanerozoic marine diversity and the fossil record. *Nature* **293**, 435–437 (1981).
3. Zaffos, A., Finnegan, S. & Peters, S. E. Plate tectonic regulation of global marine animal diversity. *Proceedings of the National Academy of Sciences* **114**, 5653–5658 (2017).
4. Valdes, P. J., Scotese, C. R. & Lunt, D. J. Deep ocean temperatures through time. *Climate of the Past* **17**, 1483–1506 (2021).
5. Grossman, E. L. & Joachimski, M. M. Ocean temperatures through the Phanerozoic reassessed. *Sci Rep* **12**, 8938 (2022).
6. Scotese, C. R. & Wright, N. PALEOMAP paleodigital elevation models (PaleoDEMS) for the Phanerozoic. *Paleomap Proj* (2018).

7. Cermeño, P. *et al.* Post-extinction recovery of the Phanerozoic oceans and biodiversity hotspots. *Nature* **607**, 507–511 (2022).

Supplementary Note 1. The different hypotheses or theories proposed to explain large-scale patterns in biodiversity.

We propose to classify the main hypotheses or theories put forward to explain large-scale biodiversity patterns such as Latitudinal Biodiversity Gradients (LBGs) into nine categories (Supplementary Fig. 1):

Category 1: Null/neutral

This first category of theories assembles together null and neutral models, which are a good starting point to any study working on large-scale biodiversity patterns to check whether observed patterns may occur by chance. A null model (Category 1.1, Supplementary Fig. 1) generates a pattern based on the randomisation of ecological data or random sampling from a known statistical distribution[1]. The most famous example of a null model in the context of biodiversity is perhaps the Mid-Domain Effect (MDE) proposed by Colwell and Hurtt[2] and described in details in Colwell and Lees[3]. This model reconstructs well the contemporary LBG by generating random species range between the two poles, demonstrating the systematic formation of a peak in biodiversity at the middle of a latitudinal range.

A neutral model (Category 1.2, Supplementary Fig. 1) is a process-based model applied at an individual level and in which birth/death and migration rates, as well as speciation/extinction rates, are neutral (i.e. individuals of all species are ecologically equivalent within a trophic guild). Examples of such models are those developed as part of the Neutral Theory of Biodiversity and Biogeography[4]. The theory has been more frequently tested at a regional than at a global scale, however[5]. Although recent developments for a neutral theory of marine plankton have been undertaken[5], the neutral theory has rarely been tested at a large scale in the oceans (see however Chust and colleagues[6]). Allen & Gillooly[7] found some support that the LBG may originate from spatial variation in speciation rate as predicted by Hubbell's theory.

Category 2: Environmental control

This category of hypotheses or theories (Category 2, Supplementary Fig. 1) invokes an environmental control of biodiversity. Temperature (Category 2.1) has frequently been suggested to explain large-scale biodiversity patterns[8,9] but the exact mechanisms by which this factor may act to create a LBG remain uncertain[10,11]. Other studies have suggested that the vertical structure of the ocean (Category 2.2) is important to understand large-scale biodiversity

patterns of plankton[12]; the stronger the vertical structure of the ocean, the greater the biodiversity. Oligotrophy (Category 2.3) has also been associated with greater biodiversity[13] and many biodiversity hotspots (e.g. coral reef ecosystems) are found in oligotrophic areas[14]. Although empirical studies have identified key environmental factors that may influence large-scale biodiversity patterns such as the LBG, they have generally failed so far to provide a theoretical framework to better understand biodiversity arrangement in space and time. Nevertheless, some parameters such as temperature have been encapsulated in theories described below.

Category 3: Energy

This third category of hypotheses or theories is based on energy (Category 3, Supplementary Fig. 1). Proposed explanations include exosomatic and endosomatic energy[15]. We also include the Metabolic Theory of Ecology (MTE) in this category because the theory uses body size (i.e endosomatic energy) and temperature (i.e exosomatic energy) to assess metabolism and related processes from the individual to the ecosystem level, including biodiversity[16]. Last, energy partitioning is included in this family as well because it relates abundance, body size and species richness[8].

Exosomatic energy theories (Category 3.1), also known as ambient energy theories, consider that biodiversity is controlled by the amount of energy available regionally[17], which is strongly modulated by climate (e.g. sunlight exposure, photoperiod, photosynthetically active radiation, temperature, water availability, potential annual evapotranspiration[15,18,19]) and hydrodynamics in the marine realm[20,21]. Several theories have attempted to give an explanation about the importance of exosomatic energy on the LBG[22–24]. For example, annual Actual EvapoTranspiration (AET) could explain between 80% and 93% of the variance in animal and plant species richness patterns[18].

Endosomatic theories (Category 3.2) link the production of endosomatic energy with the total number of species[15]. Among theories, the theory of productivity[18,25,26] posits that the proportion of primary producers in the system is fundamental because it affects the total amount of organic matter through the entire food web and therefore the number of individuals and species; low endosomatic production results in a low number of species.

The Metabolic Theory of Ecology (MTE, Category 3.3) uses some key principles of allometry and kinetics to establish predictions of metabolism, biological rates and ecological processes[16]. The metabolic rate is related to (i) body size (i.e. Kleiber's Law) and (ii) temperature (i.e. Boltzmann factor or the Van't Hoff-Arrhenius relation). Metabolism is the process allowing energy and materials to be transformed within an organism and to be exchanged between the organism and the environment. Temperature influences metabolism through its effects on biological rates and biochemical reactions. Allen and colleagues[27] proposed a mechanistic framework based on the MTE to explain large-scale biodiversity patterns. Compared to most hypotheses proposed to explain biodiversity gradients, the MTE is a process-based model that is testable against field data. Allen and co-workers successfully tested the MTE against observations in the marine and terrestrial realms[27]. Subsequently, Allen and colleagues[28,29]

extended their theory by proposing that the origin and maintenance of biodiversity gradients depend upon energetic constraints of speciation-extinction dynamics. They showed that the absolute rates of DNA evolution rise exponentially with environmental temperature in the same way as individual metabolic rates.

Energy partitioning (Category 3.4) may be an important field of research to understand large-scale biodiversity patterns[8,30–34]. Energy partitioning should relate abundance, body size and species richness and explain how this varies in space and time. Some studies have provided compelling evidence of changes in dominance of cell size across ecosystems[35] and of systematic changes in the relationships between cell size and species richness[36,37]. Beaugrand and colleagues[38,39] have also shown an inverse relationship between mean size and biodiversity of calanoid copepods in the North Atlantic. How energy partitioning varies across latitudes remains an unsolved question and more research on this point is clearly needed. Environmental requirements also differ according with body size, leading to community variations in space and time[40].

Category 4: Area

A fourth category of theory is based on area. The relationship between species richness and area is the second well-known ecological pattern after the LBG[41]. This relationship has been reviewed in details by Rosenzweig[42], who proposed that De Candolle was the first to document the pattern although the idea that area could explain large-scale biodiversity patterns and especially the LBG is often attributed to Darlington[43]. Explanations rely on the fact that a larger area is more susceptible to support a large number of individuals and therefore greater species. One of the most famous theories using area to understand biodiversity is the Theory of Island Biogeography[44], which links the number of species in a given island to the area of an island, in addition to distance from mainland.

Area also plays an indirect role in the number of species by increasing habitat heterogeneity (see category 7), which enables more niches and species to co-exist[45].

Category 5: Speciation/extinction dynamics and evolution

A fifth category of explanations deals with origination/extinction dynamics and evolution.

Three models have been proposed to explain the larger number of species in the tropics. The model “Tropics as cradle”[46–49] (Category 5.1) explains the differences in biodiversity by a higher rate of origination in the tropics and a constant rate of extinction worldwide. The model “Tropics as museum” (Category 5.2) states that the rates of origination are constant for all latitudes but that tropics have lower rates of extinction. Last, the model “Tropics as casino” (or “out of the tropics”, Category 5.3)[49,50] supposes that species have a higher rate of speciation and extinction in the tropics. The differences between “Tropics as Cradle”, “Tropics as Museum” and “Tropics as Casino” have been summarised by Mittelbach[50] and also reviewed by Arita & Vásquez-Domínguez[49]. In the model “Tropics as cradle”, tropics are the only place

of origination and extinction rates are rapidly higher than speciation rates polewards. In such a situation, high-latitude environments become rapidly unsaturated in species and mostly contain old species as the rate of speciation is low. In the model "Tropics as museum", young species dominate in high latitudes and a strong turnover can be observed due to a higher extinction rate polewards. In this model, tropics are the only place where old species can be observed because of a lower extinction rate. In the model "Tropics as casino", species origination takes place in the tropics and new species progressively move from the tropics to temperate and polar regions. In temperate regions, speciation and extinction rates balance themselves, leading to an equilibrium; community turnover is greater. In polar regions, extinction tends to become higher than speciation and more species disappear, explaining the lower number of species in these areas.

Gillooly and colleagues[51] provided evidence that the molecular clock varies with metabolism, which is itself a function of body mass and temperature (i.e. Metabolic Theory of Ecology, see also Category 3.3). Evolutionary rates (Category 5.4) should therefore be higher in low latitudes for species with similar body size. Such predictions agree with the observations that many taxonomic groups originate in the tropics[52] and that strong positive correlations have been found between the LBG and speciation for marine plankton (e.g. Radiolaria, Foraminifera and nanoplankton)[7].

Category 6: Time/history

The sixth category gathers time and history explanations. Time is an important parameter because it is needed for speciation and for species saturation of communities by dispersal. Ecological time has been distinguished from evolutionary time.

Ecological time (Category 6.1) explains the lack of species in a given area by an insufficient time for a species to move from an area to another, which depends upon processes such as dispersal capability[53]; the species already exists but has not colonised or recolonised an area yet. Ecological time supposes that a longer time in the tropics enables more species to coexist, which could explain in part the LGB. Although ecological time is not considered to be the primary factor of the LBG, it sometimes explains why some areas of similar latitudes have different species richness[53,54].

Evolutionary time (Category 6.2) is related to speciation because evolutionary time is needed for speciation to operate[53,55] (see Category 5.4).

The time stability hypothesis[56–59] (Category 6.3) states that the frequency of disturbances may strongly influence regional species richness. When the environment is stable, less energy is required for homeostasis and production of organic matter rises. This increases individuals and therefore the number of species, which become more stenoecious or specialised[60,61]. As tropics have been less disturbed, they are expected to hold more species than higher latitudes, which may explain the LGB.

Among historical hypotheses or theories to explain LBGs, Tropical Niche Conservatism (TNC, Category 6.4) proposes that there are more tropical species because most taxonomic groups

originate from the tropics and that Phylogenetic Niche Conservatism (PNC) limits the dispersal of the species of a taxonomic group outside the tropics[62]; niche evolution is therefore limited with respect to PNC. The LBG is therefore the result of the history of origination of a species combined with poor niche evolution.

Last, historical biogeography (Category 6.5) relates many macroecological patterns to history (e.g. continental drift and its influence on life distribution, vicariance events)[63].

Category 7: Habitat features

Habitat features may play an important role in LBGs[44,45,64,65]. Among explanations including habitat features, Spatial Environmental Heterogeneity (SEH, Category 7.1) is often mentioned[25,45,60,64,66–76]. Many terms have been used to qualify SEH (e.g. patchiness, habitat heterogeneity, habitat complexity, landscape structure, environmental variability)[76]. SEH generally increases with area, which inflates the number of niches (abiotic or biotic), and in turn, species richness; this is especially evident in insular biogeography[45]. SEH may also promote speciation through isolation and specialisation[76]. Although SEH is not seen as a key candidate to explain LBGs[77], it may be a key driver of changes in global species richness through geological time.

Temporal stability (Category 7.2) is an important property of a habitat. It has been frequently suggested that the great biodiversity of tropical forest and coral reefs may be explained by environmental stability[78]; proposed explanations have been summarised above (Category 6.3).

The Intermediate Disturbance Hypothesis (IDH, Category 7.3) stipulates that biodiversity is maximum in a balance between competition and selection, in other words at the middle of environmental gradients, and that species richness should be greater in habitats where environmental disturbances are intermediate in terms of frequencies and intensities[79]. While the idea has first been introduced by Grime[80] and Horn[81], this idea has been popularized by Connell[82] to explain biodiversity patterns in coral reefs.

Habitat harshness (Category 7.4) may also affect large-scale spatial biodiversity patterns[83–85]. As harshness is more important in polar and temperate regions, it may limit the number of species that can establish in a given area of the extra-tropical regions and modify their dynamics, leading to more species in the tropics.

Category 8: Niche-based theories

Category 8 gathers together niche-based theories or explanations.

Niche partitioning (Category 8.1) is the process by which species tends to evolve by occupying available niche space[86,87]. Darwin's finches on Galapagos islands is a great illustration of this process[88]. Although this theory refers more frequently to resource partitioning, it is also relevant for Hutchinson's niche partitioning. Great Spatial Environmental Heterogeneity (SEH, Category 7.1) and resource diversity may promote niche partitioning.

Niche assembly (Category 8.2) is a theory that proposes that biodiversity is higher over low latitudes because the number of niches is greater[89]. This higher number of niches is explained either by smaller niches (i.e. higher degree of stenoecy) or by greater niche overlapping. Another explanation is that the environmental gradients are larger in the tropics, enabling more niches to coexist[90]. The Niche-Assembly theory is in general based on the trophic niche[89].

Stevens[91] proposed that an augmentation in species richness towards the equator may be the result of Rapoport's rule (Category 8.3) which states that the mean latitudinal range of a species rises with latitude. The corollary of such a proposition is that a greater number of species should occur in the tropics because they are characterised by more niche overlapping. Latitudinal patterns in niche breadth have not been confirmed entirely[92] and some authors have only confirmed partially this pattern (i.e from the equator to cold-temperate marine biomes) because polar regions are characterised by lower changes in key environmental parameters such as sea temperature[93].

Niche saturation (Category 8.4)[94,95] stipulates that ecological niches are more saturated in the tropics than towards the poles. Repeated glaciations during the Pleistocene[96] have led to niche desaturation at high latitudes[97]. This explanation can be combined with the models "Tropics as cradle" (Category 5.1) and "Tropics as Museum" (Category 5.2).

Traits (Category 8.5) also affect species ability to survive in various environments, and influence communities establishing in different contexts. For example, species limitation in dispersal capabilities affect species distribution. Kléparski and colleagues[40] showed that North Sea diatoms undergo strong morphological changes throughout the year and that species with similar phenology possess comparable morphological traits (e.g. cell elongation) and ecological niches. It remains unsolved how changes in species traits may influence large-scale biodiversity patterns, however[98].

Finally, the METAL (MacroEcological Theory on the Arrangement of Life) theory (Category 8.6) uses Hutchinson's niche concept[99] and some other fundamental principles (see Methods) to reconstruct biodiversity through space and time[54,90,93,100–102]. This theory considers that the niche-environment interaction is fundamental to understand the arrangement of biodiversity from the individual to the species and community organisational level. Species richness is greater in the tropics where the number of niches is higher. Niche saturation can affect LBG patterns but moderately[93]. According to this theory, the niche-environment interaction generates a mathematical constraint on the large-scale biodiversity distribution, which also explains changes in the global pool of species richness over time and why there are more terrestrial than marine species[102,103]. The METAL theory suggests the existence of a species carrying capacity[94].

Category 9: Interspecific interaction

Category 9 gathers explanations based on interspecific interaction.

Interspecific interactions have been classified into two main categories. First, bottom-up control (Category 9.1) considers competition to negatively influence biodiversity. Speciation may also

depend on species richness itself and Chen & He[104] showed that endemic species increases with species richness on islands. It is also possible that high species diversity propagates from low to high trophic levels, more resource diversity promoting more diverse consumers and predators[90] (see Category 8.1). Conversely, top-down control (Category 9.2) enhances biodiversity by alleviating prey competition and therefore enabling more species co-existence. The competition-predation trade-off theory (Category 9.3) considers the balance between top-down and bottom-up controls being fundamental in the maintenance of species richness locally[105]. Although these interactions might affect biodiversity locally, it is difficult to envision how they may control large-scale biodiversity patterns, including LBGs[53,90].

SUPPLEMENTARY NOTE REFERENCES

1. Gotelli, N. J. & Graves, G. R. *Null Models in Ecology*. (1996).

2. Colwell, R. K. & Hurtt, G. C. Nonbiological gradients in species richness and a spurious rapoport effect. *The American Naturalist* **144**, 570–595 (1994).

3. Colwell, R. K. & Lees, D. C. The mid-domain effect: geometric constraints on the geography of species richness. *Trends in Ecology & Evolution* **15**, 70–76 (2000).

4. Hubbell, S. P. *The Unified Neutral Theory of Biodiversity and Biogeography*. (Princeton University Press, Princeton, 2001).

5. Behrenfeld, M. J. & Bisson, K. M. Neutral Theory and Plankton Biodiversity. *Annual Review of Marine Science* **16**, 283–305 (2024).

6. Chust, G., Irigoien, X., Chave, J. & Harris, R. P. Latitudinal phytoplankton distribution and the neutral theory of biodiversity. *Global Ecology and Biogeography* **22**, 531–543 (2013).

7. Allen, A. P. & Gillooly, J. F. Assessing latitudinal gradients in speciation rates and biodiversity at the global scale. *Ecology Letters* **9**, 947–954 (2006).

8. Beaugrand, G., Edwards, M. & Legendre, L. Marine biodiversity, ecosystem functioning, and carbon cycles. *Proc. Natl. Acad. Sci. U.S.A.* **107**, 10120–10124 (2010).

9. Tittensor, D. P. *et al.* Global patterns and predictors of marine biodiversity across taxa. *Nature* **466**, 1098–1101 (2010).

10. Currie, D. J. *et al.* Predictions and tests of climate-based hypotheses of broad-scale variation in taxonomic richness. *Ecology Letters* **7**, 1121–1134 (2004).

11. Amorim, F. de L. L. de *et al.* Investigation of marine temperature changes across temporal and spatial Gradients: Providing a fundament for studies on the effects of warming on marine ecosystem function and biodiversity. *Progress in Oceanography* **216**, 103080 (2023).

12. Rutherford, S., D'Hondt, S. & Prell, W. Environmental controls on the geographic distribution of zooplankton diversity. *Nature* **400**, 749–753 (1999).

13. Rombouts, I. *et al.* Global latitudinal variations in marine copepod diversity and environmental factors. *Proc. R. Soc. B: Biol. Sci.* **276**, 3053–3062 (2009).

14. Maragos, J. E., Crosby, M. P. & McManus, J. W. Coral reefs and biodiversity: a critical and threatened relationship. *Oceanography* **9**, 83–99 (1996).

15. Hawkins, B. A., Porter, E. E. & Felizola Diniz-Filho, J. A. Productivity and history as predictors of the latitudinal diversity gradient of terrestrial birds. *Ecology* **84**, 1608–1623 (2003).

16. Brown, J. H., Gillooly, J. F., Allen, A. P., Savage, V. M. & West, G. B. Toward a metabolic theory of ecology. *Ecology* **85**, 1771–1789 (2004).

17. O'Brien, E. M., Field, R. & Whittaker, R. J. Climatic gradients in woody plant (tree and shrub) diversity: water-energy dynamics, residual variation, and topography. *Oikos* **89**, 588–600 (2000).

18. Currie, D. J. Energy and large-scale patterns of animal- and plant-species richness. *The American Naturalist* **137**, 27–49 (1991).

19. Alekseev, V. V. Model of the number of species in an ecosystem in dependence on geographical latitude. *Ecological Modelling* **17**, 107–112 (1982).

20. Ruddiman, W. F. Recent planktonic foraminifera: dominance and diversity in North Atlantic surface sediments. *Science* **164**, 1164–1167 (1969).

21. Beaugrand, G., Reid, P. C., Ibanez, F., Lindley, J. A. & Edwards, M. Reorganization of North Atlantic marine copepod biodiversity and climate. *Science* **296**, 1692–1694 (2002).

22. Tittensor, D. P. *et al.* Global patterns and predictors of marine biodiversity across taxa. *Nature* **466**, 1098–1101 (2010).

23. Rutherford, S., D'Hondt, S. & Prell, W. Environmental controls on the geographic distribution of zooplankton diversity. *Nature* **400**, 749–753 (1999).

24. Beaugrand, G., Reid, P., Ibañez, F., Lindley, J. & Edwards, M. Reorganization of North Atlantic Marine Copepod Biodiversity and Climate. *Science (New York, N.Y.)* **296**, 1692–4 (2002).

25. Pianka, E. R. Latitudinal gradients in species diversity: a review of concepts. *The American Naturalist* **100**, 33–46 (1966).

26. MacArthur, R. H. Patterns of communities in the tropics. *Biological Journal of the Linnean Society* **1**, 19–30 (1969).

27. Allen, A. P., Brown, J. H. & Gillooly, J. F. Global Biodiversity, Biochemical Kinetics, and the Energetic-Equivalence Rule. *Science* **297**, 1545–1548 (2002).

28. Allen, A. P., Gillooly, J. F., Savage, V. M. & Brown, J. H. Kinetic effects of temperature on rates of genetic divergence and speciation. *Proceedings of the National Academy of Sciences* **103**, 9130–9135 (2006).

29. Allen, A. P., Gillooly, J. F. & Brown, J. H. Recasting the species–energy hypothesis: the different roles of kinetic and potential energy in regulating biodiversity. in *Scaling Biodiversity* (eds. Storch, D., Brown, J. & Marquet, P.) 283–299 (Cambridge University Press, Cambridge, 2007). doi:10.1017/CBO9780511814938.016.

30. Lawton, J. H., Hassell, M. P. & May, R. M. Species richness and population dynamics of animal assemblages. Patterns in body size: abundance space. *Philosophical Transactions of the Royal Society of London. Series B: Biological Sciences* **330**, 283–291 (1997).

31. Sieburth, J. McN., Smetacek, V. & Lenz, J. Pelagic ecosystem structure: Heterotrophic compartments of the plankton and their relationship to plankton size fractions 1. *Limnol. Oceanogr.* **23**, 1256–1263 (1978).

32. Li, W. K. W. Macroecological patterns of phytoplankton in the northwestern North Atlantic Ocean. *Nature* **419**, 154–157 (2002).

33. Li, B.-L., Gorshkov, V. G. & Makarieva, A. M. Energy Partitioning between Different-Sized Organisms and Ecosystem Stability. *Ecology* **85**, 1811–1813 (2004).

34. Beaugrand, G. Decadal changes in climate and ecosystems in the North Atlantic Ocean and adjacent seas. *Deep Sea Res. Part II Top. Stud. Oceanogr.* **56**, 656–673 (2009).

35. Li, B.-L., Gorshkov, V. G. & Makarieva, A. M. Energy Partitioning between Different-Sized Organisms and Ecosystem Stability. *Ecology* **85**, 1811–1813 (2004).

36. Li, W. K. W. Macroecological patterns of phytoplankton in the northwestern North Atlantic Ocean. *Nature* **419**, 154–157 (2002).

37. Cermeño, P. & Figueiras, F. G. Species richness and cell-size distribution: size structure of phytoplankton communities. *Marine Ecology Progress Series* **357**, 79–85 (2008).

38. Beaugrand, G., Edwards, M. & Legendre, L. Marine biodiversity, ecosystem functioning, and carbon cycles. *Proceedings of the National Academy of Sciences* **107**, 10120–10124 (2010).

39. Beaugrand, G. Decadal changes in climate and ecosystems in the North Atlantic Ocean and adjacent seas. *Deep Sea Research Part II: Topical Studies in Oceanography* **56**, 656–673 (2009).

40. Kléparski, L. *et al.* Morphological traits, niche-environment interaction and temporal changes in diatoms. *Prog. Oceanogr.* **201**, 102747 (2022).

41. Hawkins, B. A. & Porter, E. E. Area and the latitudinal diversity gradient for terrestrial birds. *Ecology Letters* **4**, 595–601 (2001).

42. Rosenzweig, M. L. *Species Diversity in Space and Time*. (Cambridge University Press, Cambridge/Cambridge University Press, 1995).

43. Darlington, P. J. J. *Zoogeography: The Geographical Distribution of Animals*. (John Wiley & Sons, 1966).

44. MacArthur, R. H. & Wilson, E. O. *The Theory of Island Biogeography*. (Princeton University Press, 1967).

45. Hortal, J., Triantis, K. A., Meiri, S., Thébault, E. & Sfenthourakis, S. Island species richness increases with habitat diversity. *The American Naturalist* **174**, E205–E217 (2009).

46. Stebbins, G. L. Building bridges between evolutionary disciplines. *Taxon* **23**, 11–20 (1974).

47. Chown, S. L. & Gaston, K. J. Areas, cradles and museums: the latitudinal gradient in species richness. *Trends in Ecology & Evolution* **15**, 311–315 (2000).

48. Jablonski, D., Roy, K. & Valentine, J. W. Out of the tropics: evolutionary dynamics of the latitudinal diversity gradient. *Science* **314**, 102–106 (2006).

49. Arita, H. T. & Vázquez-Domínguez, E. The tropics: cradle, museum or casino? A dynamic null model for latitudinal gradients of species diversity. *Ecology Letters* **11**, 653–663 (2008).

50. Mittelbach, G. G. *et al.* Evolution and the latitudinal diversity gradient: speciation, extinction and biogeography. *Ecology Letters* **10**, 315–331 (2007).

51. Gillooly, J. F., Allen, A. P., West, G. B. & Brown, J. H. The rate of DNA evolution: Effects of body size and temperature on the molecular clock. *Proceedings of the National Academy of Sciences* **102**, 140–145 (2005).

52. Jablonski, D. The tropics as a source of evolutionary novelty through geological time. *Nature* **364**, 142–144 (1993).

53. Rohde, K. Latitudinal gradients in species diversity: the search for the primary cause. *Oikos* **65**, 514 (1992).

54. Beaugrand, G., Luczak, C., Goberville, E. & Kirby, R. R. Marine biodiversity and the chessboard of life. *PLOS ONE* **13**, e0194006 (2018).

55. Pianka, E. R. *Evolutionary Ecology*. (1974).

56. Wallace, A. R. *Tropical Nature, and Other Essays*. (Macmillan and Company, 1878).

57. Fischer, A. G. Latitudinal Variations in Organic Diversity. *Evolution* **14**, 64–81 (1960).

58. Simpson, G. G. Species Density of North American Recent Mammals. *Systematic Zoology* **13**, 57–73 (1964).

59. Haffer, J. Speciation in Amazonian Forest Birds. *Science* **165**, 131–137 (1969).

60. Connell, J. H. & Orias, E. The Ecological Regulation of Species Diversity. *The American Naturalist* **98**, 399–414 (1964).

61. Abele, L. G. & Walters, K. The Stability-Time Hypothesis: Reevaluation of the Data. *The American Naturalist* **114**, 559–568 (1979).

62. Wiens, J. J. & Donoghue, M. J. Historical biogeography, ecology and species richness. *Trends in Ecology & Evolution* **19**, 639–644 (2004).

63. Lomolino, M. V., Brown, J. H. & Sax, D. F. Island Biogeography Theory: Reticulations and Reintegration of "a Biogeography of the Species". in *The Theory of Island Biogeography Revisited* (eds. Losos, J. B. & Ricklefs, R. E.) 13–51 (Princeton University Press, 2009). doi:10.1515/9781400831920.13.

64. Kerr, J. T. & Packer, L. Habitat heterogeneity as a determinant of mammal species richness in high-energy regions. *Nature* **385**, 252–254 (1997).

65. Beaugrand, G., Reid, P. C., Ibañez, F. & Planque, B. Biodiversity of North Atlantic and North Sea calanoid copepods. *Mar. Ecol. Prog. Ser.* **204**, 299–303 (2000).

66. MacArthur, R. H. & MacArthur, J. W. On Bird Species Diversity. *Ecology* **42**, 594–598 (1961).

67. Klopfer, P. H. Environmental Determinants of Faunal Diversity. *The American Naturalist* **93**, 337–342 (1959).

68. Klopfer, P. H. & MacArthur, R. H. On the Causes of Tropical Species Diversity: Niche Overlap. *The American Naturalist* **95**, 223–226 (1961).

69. Macarthur, R. H. Patterns of Species Diversity. *Biological Reviews* **40**, 510–533 (1965).

70. Margalef, R. Diversity and stability: a practical proposal and a model of interdependence. (1969).

71. Huston, M. A General Hypothesis of Species Diversity. *The American Naturalist* **113**, 81–101 (1979).

72. Thiery, R. G. Environmental Instability and Community Diversity*. *Biological Reviews* **57**, 691–710 (1982).

73. Shmida, A. & Wilson, M. V. Biological Determinants of Species Diversity. *Journal of Biogeography* **12**, 1–20 (1985).

74. Staff, G. M. & Powell, E. N. The paleoecological significance of diversity: The effect of time averaging and differential preservation on macroinvertebrate species richness in death assemblages. *Palaeogeography, Palaeoclimatology, Palaeoecology* **63**, 73–89 (1988).

75. Kolasa, J. & Rollo, C. D. Introduction: The Heterogeneity of Heterogeneity: A Glossary. in *Ecological Heterogeneity* (eds. Kolasa, J. & Pickett, S. T. A.) 1–23 (Springer, New York, NY, 1991). doi:10.1007/978-1-4612-3062-5_1.

76. Stein, A. & Kreft, H. Terminology and quantification of environmental heterogeneity in species-richness research. *Biological Reviews* **90**, 815–836 (2015).

77. Triantis, K. A., Mylonas, M., Lika, K. & Vardinoyannis, K. A model for the species–area–habitat relationship. *Journal of Biogeography* **30**, 19–27 (2003).

78. Connell, J. H. & Orias, E. The Ecological Regulation of Species Diversity. *The American Naturalist* (1964) doi:10.1086/282335.

79. Moi, D. A., García-Ríos, R., Hong, Z., Daquila, B. V. & Mormul, R. P. Intermediate Disturbance Hypothesis in Ecology: A Literature Review. *anzf* **57**, 67–78 (2020).

80. Grime, J. P. Control of species density in herbaceous vegetation. (1973).

81. Horn, H. S. Markovian properties of forest succession. *Ecology and evolution of communities* 196–211 (1975).

82. Connell, J. H. Diversity in Tropical Rain Forests and Coral Reefs. *Science* **199**, 1302–1310 (1978).

83. Brown, J. H. & Gibson, A. C. *Biogeography*. (Mosby, 1983).

84. Begon, M., Harper, J. L. & Townsend, C. R. *Ecology. Individuals, Populations and Communities.* (Blackwell scientific publications, 1986).

85. Thiery, R. G. Environmental instability and community diversity. *Biological Reviews* **57**, 691–710 (1982).

86. MacArthur, R. H. Population Ecology of Some Warblers of Northeastern Coniferous Forests. *Ecology* **39**, 599–619 (1958).

87. Hector, A. & Hooper, R. Darwin and the First Ecological Experiment. *Science* **295**, 639–640 (2002).

88. Grant, B. R. & Grant, P. R. Fission and fusion of Darwin's finches populations. *Philosophical Transactions of the Royal Society B: Biological Sciences* **363**, 2821–2829 (2008).

89. Turner, J. R. G. Explaining the global biodiversity gradient: energy, area, history and natural selection. *Basic and Applied Ecology* **5**, 435–448 (2004).

90. Beaugrand, G. *Marine Biodiversity, Climatic Variability and Global Change*. (Routledge, London, 2015).

91. Stevens, G. C. The latitudinal gradient in geographical range: how so many species coexist in the tropics. *Am. Nat.* **133**, 240–256 (1989).

92. Vázquez, D. P. & Stevens, R. D. The Latitudinal Gradient in Niche Breadth: Concepts and Evidence. *The American Naturalist* **164**, E1–E19 (2004).

93. Beaugrand, G., Rombouts, I. & Kirby, R. R. Towards an understanding of the pattern of biodiversity in the oceans: The pattern of biodiversity in the oceans. *Glob. Ecol. Biogeogr.* **22**, 440–449 (2013).

94. Storch, D. & Okie, J. G. The carrying capacity for species richness. *Global Ecology and Biogeography* **28**, 1519–1532 (2019).

95. Pontarp, M. *et al.* The latitudinal diversity gradient: novel understanding through mechanistic eco-evolutionary models. *Trends in Ecology & Evolution* **34**, 211–223 (2019).

96. Fischer, A. G. Latitudinal Variations in Organic Diversity. *Evolution* **14**, 64–81 (1960).

97. Rohde, K. *Nonequilibrium Ecology*. (Cambridge University Press, 2006).

98. Biswas, S. R. *et al.* Putting space into trait ecology: Trait, environment and biodiversity relationships at multiple spatial scales. *Journal of Ecology* **112**, 613–628 (2024).

99. Hutchinson, G. E. Concluding remarks. *Cold Spring Harb. Symp. Quant. Biol.* **22**, 415–427 (1957).

100. Beaugrand, G., Edwards, M., Raybaud, V., Goberville, E. & Kirby, R. R. Future vulnerability of marine biodiversity compared with contemporary and past changes. *Nat. Clim. Chang.* **5**, 695–701 (2015).

101. Beaugrand, G., Kirby, R. R. & Goberville, E. The mathematical influence on global patterns of biodiversity. *Ecol. Evol.* **10**, 6494–6511 (2020).

102. Beaugrand, G. Towards an understanding of large-scale biodiversity patterns on land and in the sea. *Biology* **12**, 339 (2023).

103. Ontiveros, D. E. *et al.* Impact of global climate cooling on Ordovician marine biodiversity. *Nat Commun* **14**, 6098 (2023).

104. Chen, X.-Y. & He, F. Speciation and endemism under the model of island biogeography. *Ecology* **90**, 39–45 (2009).

105. Terborgh, J. W. Toward a trophic theory of species diversity. *Proceedings of the National Academy of Sciences* **112**, 11415–11422 (2015).
