# Supplementary material for: Unravelling the drivers of marine biodiversity across the Phanerozoic
Source: Nat Commun. 2025 Sep 26;16:8498. doi: 10.1038/s41467-025-63428-9 (PMC12475154; doi:10.1038/s41467-025-63428-9)
Supplement: Supplementary file 2 — Description of Addtional Supplementary File [file 41467_2025_63428_MOESM2_ESM.pdf]

Description of Additional Supplementary File

Supplementary movie 1. Global maps of simulated marine biodiversity during the Phanerozoic. Robinson projection with parallels shown every 30° latitude. Emerged landmasses are shaded gray.
